# Migration and tuberculosis transmission in a middle-income country: a cross-sectional study in a central area of São Paulo, Brazil

**DOI:** 10.1186/s12916-018-1055-1

**Published:** 2018-04-30

**Authors:** Julia Moreira Pescarini, Vera Simonsen, Lucilaine Ferrazoli, Laura C. Rodrigues, Rosangela S. Oliveira, Eliseu Alves Waldman, Rein Houben

**Affiliations:** 1 Faculdade de Saúde Pública, Universidade de São Paulo, São Paulo, Brazil; 2 Instituto Adolfo Lutz, São Paulo, Brazil; 3 Department of Infectious Disease Epidemiology, Faculty of Epidemiology and Public Health, London School of Hygiene and Tropical Medicine, London, UK; 4 TB Modelling Group, TB Centre, London School of Hygiene and Tropical Medicine, London, UK

**Keywords:** Tuberculosis, Molecular epidemiology, Transmission, Migration, Middle-income; disease control

## Abstract

**Background:**

Little is known about the impact of growing migration on the pattern of tuberculosis (TB) transmission in middle-income countries. We estimated TB recent transmission and its associated factors and investigated the presence of cross-transmission between South American migrants and Brazilians.

**Methods:**

We studied a convenient sample of cases of people with pulmonary TB in a central area of São Paulo, Brazil, diagnosed between 2013 and 2014. Cases with similar restriction fragment length polymorphism (IS*6110*-RFLP) patterns of their *Mycobacterium tuberculosis* complex isolates were grouped in clusters (recent transmission). Clusters with both Brazilian and South American migrants were considered mixed (cross-transmission). Risk factors for recent transmission were studied using logistic regression.

**Results:**

Isolates from 347 cases were included, 76.7% from Brazilians and 23.3% from South American migrants. Fifty clusters were identified, which included 43% South American migrants and 60.2% Brazilians (odds ratio = 0.50, 95% confidence interval = 0.30–0.83). Twelve cross-transmission clusters were identified, involving 24.6% of all clustered cases and 13.8% of all genotyped cases, with migrants accounting for either an equal part or fewer cases in 11/12 mixed clusters.

**Conclusions:**

Our results suggest that TB disease following recent transmission is more common among Brazilians, especially among those belonging to high-risk groups, such as drug users. Cross-transmission between migrants and Brazilians was present, but we found limited contributions from migrants to Brazilians in central areas of São Paulo and vice versa.

**Electronic supplementary material:**

The online version of this article (10.1186/s12916-018-1055-1) contains supplementary material, which is available to authorized users.

## Background

Tuberculosis (TB) in high-income countries is often driven by migration from countries with a higher TB burden, which can account for up to 80% of the total TB burden [1]. As a result, migrants tend to be seen as a potential source of transmission to the local-born population [2, 3]. However, molecular research from high-income countries has shown that transmission from migrants to the local-born population is often limited [4]. As former high burden countries in the Global South grow economically and make progress in TB control, they are facing a similar challenge through increasing South-South migration [5].

Molecular epidemiology studies can estimate the number of TB cases due to recent transmission between local-born and migrant populations (cross-transmission) [6]. Results from high-income countries suggest that cross-transmission is bidirectional, limited and has wide variation among study settings [7–9]. There is little evidence available from middle-income countries [10, 11], where *Mycobacterium tuberculosis* complex (*Mtbc)* transmission between migrants and local-born people, in the South-South migration context, might be more pronounced because of increased mixing through cultural proximity and social integration [12].

Regional migration has increased significantly in South America over the past 15 years, with a predominance of young, healthy and more feminized populations involved in labour migration towards large urban centres in Argentina, Brazil and Chile [13]. While regional migration in South America can contribute to more social integration, the vulnerability of migrants can be enhanced by the already poor social context found in metropolitan areas of South America [14–16]. The vulnerability of the local-born population and the contexts in which migrants find themselves might contribute to more TB cross-transmission than what is evidenced in high-income countries [4, 12, 13].

While the World Health Organization (WHO) End TB Strategy recognizes migrants as one of the vulnerable populations who must be targeted [17], many middle-income countries do not have specific TB policies for internal or external migrants [18–20]. For this to change, evidence is needed to establish whether migration is contributing to the TB burden in middle-income countries and to ongoing transmission among the local population. Here, we estimated recent transmission of TB and its associated factors and investigated the presence of cross-transmission between South American migrants and Brazilians in central districts of the city of São Paulo.

## Methods

### Study design, area and population

We conducted a cross-sectional study in the municipality of São Paulo, Brazil. The Brazilian Health System (SUS) guarantees access to free and universal healthcare services, irrespective of country of origin [19], which is important for TB treatment completion and cure among migrants [21]. Migration flows in SP are predominantly from other parts of Brazil and other South American countries. The number of TB notifications among South American migrants has increased in the last ten years [21]. Migrants coming from Bolivia accounted for almost half of the notified cases in some city districts [21], and this probably reflects the three times higher yearly incidence of TB in Bolivia than in Brazil (117/100,00 inhabitants/year and 41 inhabitants/year in 2015) [22].

**Fig. 1:**
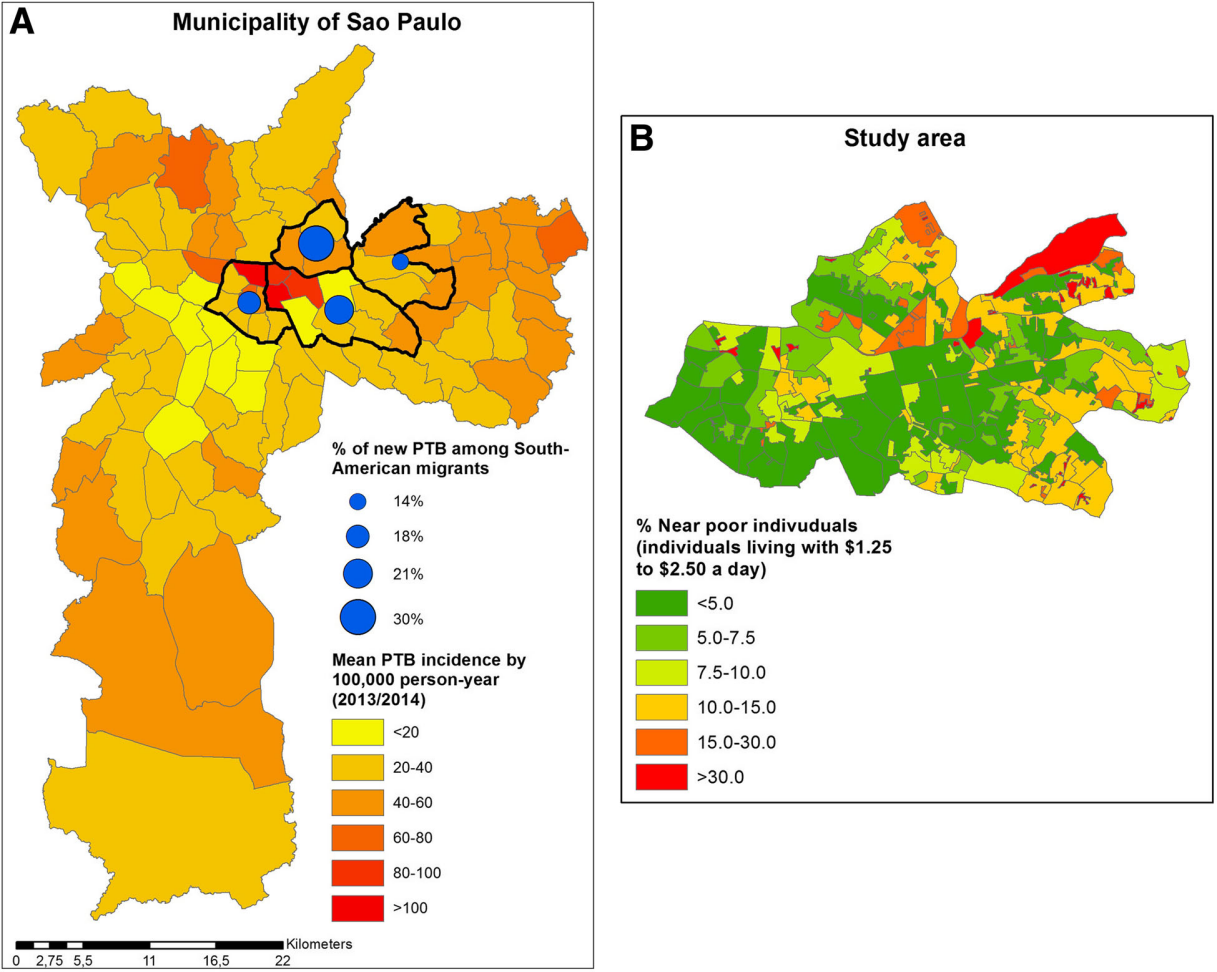
**a** Yearly mean incidence of pulmonary tuberculosis (*PTB*) among residents in the city of São Paulo for 2013/2014 and percentage of South American migrants in the study area according to the four Administrative Regions studied. **b** Near poor individuals living in the study area

Our study focused on the central area of São Paulo, where vulnerable populations including a significant number of recently arrived migrants live. We selected four Administrative Regions (administrative division of the municipality based on grouped districts) with the highest absolute number of cases of TB among South American migrants (study area). Almost 2 million individuals live in this densely populated study area (11,934 residents/km^2^), where the proportion of *near poor*^1^ reached more than 30% in some regions in 2010 (see Fig. 1) [23]. Many live in a combination of squatting and informal dwellings, including migrants under precarious work conditions living in the workplace [24]. The mean pulmonary TB (PTB) incidence rate for 2013/2014 ranged from 13 to 131/100,000 inhabitants/year in the districts, and the proportion of new PTB cases among individuals of South American origin in 2013 and 2014 ranged from 14% to 30% in each of the Administrative Regions studied. In this context, TB transmission is favourable in areas of overcrowding, poverty and inequality [17].

The Brazilian TB Control Programme recommends further confirmation by both culture and drug susceptibility test for TB cases in high-risk groups for TB. This includes people living with HIV (PLHIV), drug users, people in contact with drug-resistant TB cases, smear-positive cases after 2 months on TB treatment, retreatment TB cases and other members of the ‘vulnerable population’ including migrants from South American countries [19, 25].

Our reference population was all PTB cases among residents in the study area, and the study population included only patients with TB for whom there was a culture available, without age or sex restrictions. All *Mtb*c culture-positive PTB cases among Brazilians and migrants, available at the State Reference Laboratory for TB (Instituto Adolfo Lutz-São Paulo (IAL-SP)) between 1 January 2013 and 31 December 2014, were eligible for the study. We included only the first available respiratory sample with culture (including sputum and bronchoalveolar lavage) from all the individuals and excluded migrants from outside South America.

### Data collection

Socio-demographic, clinical and epidemiological data were obtained from the São Paulo Tuberculosis Control Program database (SINAN-TBWEB) and laboratory information from the IAL-SP database (SIGH).

### Molecular characterization

We performed the molecular characterization of *Mtbc* isolates by restriction fragment length polymorphism (IS*6110*-RFLP) at the IAL-SP [26]. Analysis of the *Mtbc* pattern was conducted in Bionumerics v.7.2 (Applied Maths, Kortrijk, Belgium). We excluded similar patterns with less than five bands.

### Data analysis and definitions

Cases with a unique IS*6110*-RFLP pattern were identified and considered to result from reactivation, not in a transmission chain in the sample. Two or more cases with identical patterns or with one band difference were defined as a cluster (i.e. part of the same recent transmission chain). Clusters consisting of either all Brazilian or all South American migrant cases were defined as simple clusters, and those with at least one Brazilian and one South American migrant were defined as mixed clusters. Statistical analyses were conducted in Stata 14.1. Specific analyses included the following:

1. *Descriptive analysis of the population*. We described the socio-demographic and epidemiological characteristics of all the TB cases in the studied area (reference population) and in the studied sample, stratified by origin (Brazilians or other South American migrants), in order to characterize Brazilians and migrants with PTB in our sample and to identify potential selection bias resulting from overrepresentation of potential high-risk populations for recent transmission within the sample (drug users, alcohol abusers and PLHIV).
2. *Clustering analysis*. The ‘*n*’ method was used to estimate the proportion of cases involved in recent transmission in the central area of São Paulo. The alternative, the ‘*n* – 1’ method, which discounts one case from each cluster that may have occurred by disease reactivation, is vulnerable to strong underestimation of ongoing transmission when the sampling proportion is small, as was likely in our study [6, 27]. We described the simple and mixed clusters in our sample, emphasizing the proportion of clustering in Brazilians and South American migrants. We investigated the associated factors of belonging to a cluster compared to unique profiles for the studied individuals estimating the odds ratio (OR) and its 95% confidence interval (95% CI) using univariate and multiple logistic regression.
3. *Sensitivity analyses*. We estimated the proportion of clusters that would be found under a more restrictive cluster definition and considered only identical isolates as part of a cluster. A second sensitivity analysis explored the bias introduced by overrepresentation of high-risk groups. We removed all PLHIV, drug users and alcohol abusers from the second analysis to estimate the proportion of overall clusters and mixed clusters.

## Results

### Descriptive analysis of the population

**Fig. 2:**
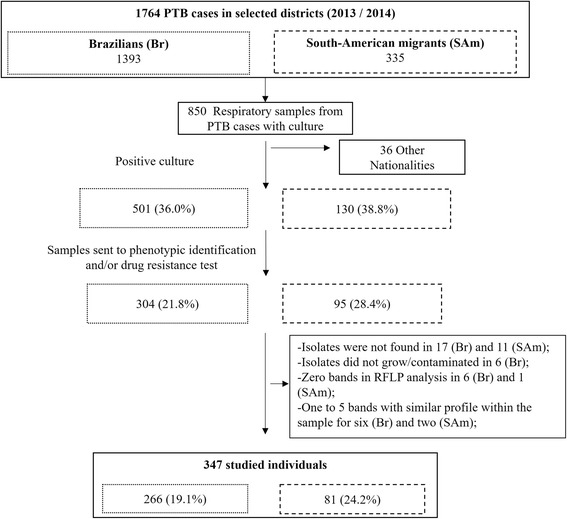
Sample selection description among notified cases in the studied area from São Paulo during 2013 and 2014

During 2013 and 2014, 1764 cases of people with PTB were reported in the study area. Approximately 79% were Brazilians, and 19% were from other South American countries. The remaining 2% (36 cases) were migrants from other regions and were excluded from this analysis. We genotyped *Mtb*c isolates of 347 cases from 631 (55%) which were culture-positive. Our sample was set by isolates from 19.7% of all cases, 266 (19.1%) Brazilian cases and 81 (24.2%) South American cases which occurred in the study area in both years (see Fig. 2).

Table 1 shows the socio-demographic and epidemiologic characteristics of the sampled PTB cases, stratified by origin. Compared to overall cases that occurred in the studied area (Additional file 1: Table S1), our sample had a higher frequency of retreatments or relapses (22.7% vs 16.7%), sputum smear-positive diagnosis (79.1% vs 72.9%) and a greater proportion of PLHIV (25.6% vs 18.4%) and drug users (16.7% vs 11.5%). We observed this trend among sampled Brazilians but not among sampled South American migrants, who shared similar characteristics with cases among South Americans in the studied area.

**Table 1:** Characteristics of PTB cases identified in the study area and in the sample, stratified by Brazilian or other South American nationalities

	Brazilians (*n* = 266)	South American migrants (*n* = 81)	Total (*n* = 347)	
	*N* (%)	*N* (%)	*N* (%)	*p* value*
Mean age in years (SD)	38.0 (13.6)	27.0 (8.9)	35.4 (13.5)	< 0.001
Sex				0.130
Male	197 (74.1)	53 (65.4)	250 (72.1)	
Female	69 (25.9)	28 (34.6)	97 (27.9)	
School attendance				0.191
0–3	17 (7.62)	3 (4.9)	20 (7.0)	
4–7	74 (33.2)	14 (22.9)	88 (31.0)	
8–11	101 (45.3)	30 (49.2)	131 (46.1)	
12+	31 (13.9)	14 (23.0)	284 (15.9)	
Case				< 0.001
New	187 (72.2)	75 (93.8)	262 (77.3)	
Retreatment/relapse	72 (27.8)	5 (6.2)	77 (22.7)	
Treatment outcome				0.318
Cure	178 (70.1)	56 (77.8)	234 (71.8)	
Loss of follow-up	60 (23.6)	11 (15.3)	71 (21.8)	
Death/failure	16 (6.3)	5 (2.8)	21 (6.4)	
Sputum smear				0.160
Negative	50 (19.2)	21 (26.6)	71 (20.9)	
Positive	210 (80.8)	58 (73.4)	268 (79.1)	
Drug resistance^a^				0.596
No	210 (87.5)	70 (89.7)	280 (88.1)	
Yes	30 (12.5)	8 (10.3)	38 (11.9)	
HIV test				< 0.001
Negative	162 (67.5)	70 (97.2)	232 (74.4)	
Positive	78 (32.5)	2 (2.8)	80 (25.6)	
Diabetes				**–**
No	251 (94.4)	81 (100)	332 (95.7)	
Yes	15 (5.6)	0 (0)	15 (4.3)	
Alcohol abuse				0.002
No	219 (82.3)	78 (96.3)	297 (85.6)	
Yes	47 (17.7)	3 (3.7)	50 (14.4)	
Drug user				< 0.001
No	210 (78.9)	79 (97.5)	289 (83.3)	
Yes	56 (21.1)	2 (2.5)	58 (16.7)	

The percentage in brackets is calculated based on non-missing data. The difference between the total number of Brazilians, South American migrants or Total and each variable category corresponds to missing data

*Two tailed *t* test used for mean age comparison and Pearson chi-square for categorical variables

^a^Resistant to at least one drug

Overall, our sample was predominantly male (72.1%), almost half of the sample attended school for 8 to 11 years (46.1%) and more than 77% were workers. The TB diagnosis was performed by active case finding for 6.4% cases; nearly 30% of all persons had five or more household contacts, and 61 of 135 individuals (30.4%) reported 12 or more weeks between disease onset and the start of drug therapy. South Americans were younger than Brazilians (mean age 27.0 vs 38.0, *p* < 0.001), and a higher proportion of them were females (34.6% vs 25.9%, *p* = 0.191). There was a lower proportion of retreatment and relapses (6.2% vs 27.8%, *p* < 0.001) and a similar cure proportion (77.8 vs 70.1%, *p* = 0.318). Among South American migrants smaller numbers of PLHIV (2.8% vs 32.5%, *p* < 0.001), alcohol abusers (3.7% vs 17.7%, *p* = 0.002) and drug users (2.5% vs 21.1%, *p* < 0.001) were observed.

### Clustering analysis

**Fig. 3:**
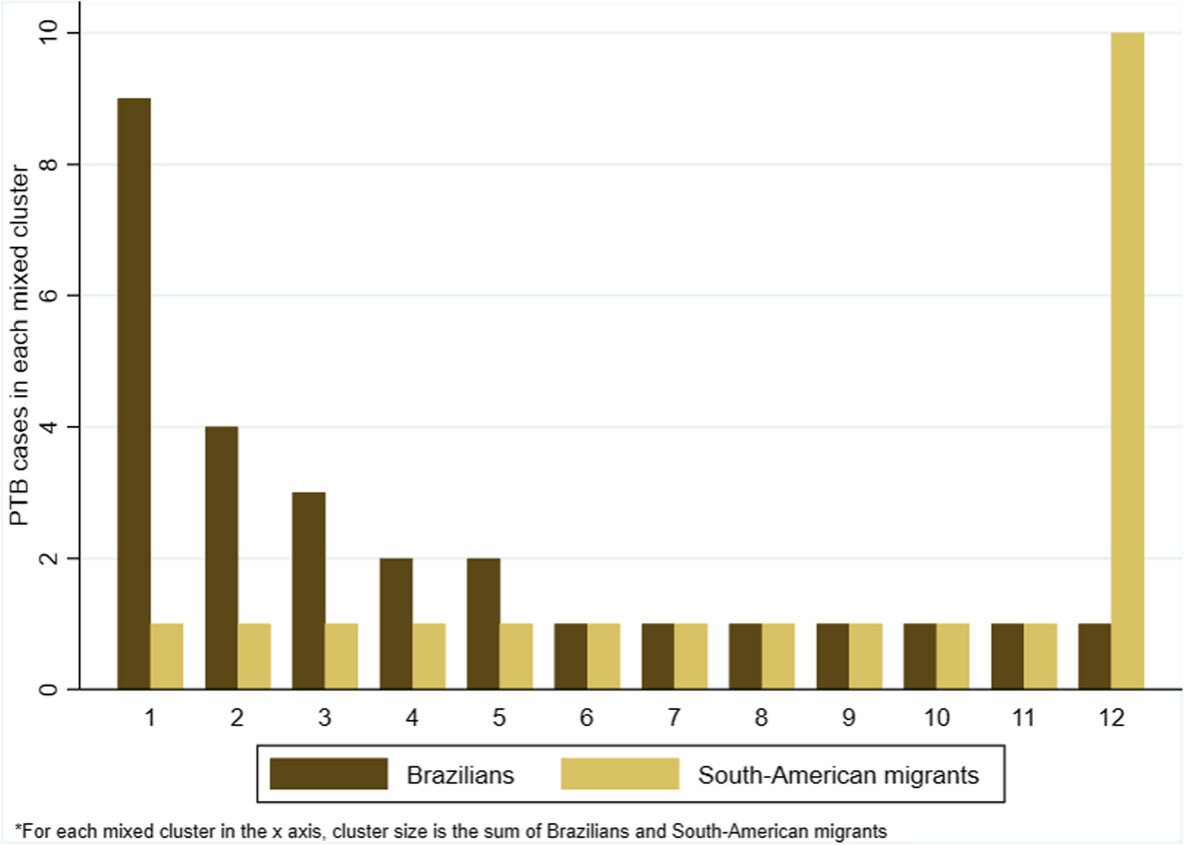
Number of individuals involved in mixed clusters. For each mixed cluster on the *x* axis, *brown* represents the number of Brazilians and *beige* the number of South American migrants

From the 347 individuals with typed *Mtb*c isolates, 152 (43.8%) had unique profiles and 195 (56.2%) were grouped in 58 clusters, of which 46 were simple and 12 were mixed. In the simple clusters, 40 contained only Brazilians and 6 only South American migrants. Simple clusters ranged from 2 to 18 individuals in Brazilians and from two to three in migrants. Forty-eight individuals were grouped in 12 mixed clusters, representing 13.8% of all genotyped cases or 24.6% of clustered cases. Figure 3 illustrates all the mixed clusters and their distribution according to their origin. Of the 12 mixed clusters, six had only two individuals — one Brazilian and one South American — five had more Brazilians than South American migrants and one had more migrants than Brazilians.

In Table 2 we compare clusters with unique patterns according to their social, demographic and clinical characteristics. South American migrants made up 23.3% (81/347) of the sample. The cluster proportion was 43.2% (35/81) among South American migrants and 60.2% (160/266) among Brazilians (OR = 0.50, 95% CI = 0.30–0.83). Overall, drug users were more likely to be part of a cluster (OR = 2.11, 95% CI = 01.15–3.89), while the proportion of HIV/TB individuals in clusters did not differ between clusters and non-clustered cases (OR = 1.56, 95% CI = 0.92–2.65). After adjustment for age, sex, case type and TB/HIV co-infection, drug users (OR adj = 1.77, 95% CI = 0.85–3.68) and being South American (OR adj = 0.66, 95% CI = 0.35–1.27) were no longer strongly linked with clustering.

**Table 2:** Distribution of cases clustered and non-clustered in our sample according to potential associated factors

Characteristics (*N*)	Clusters (*n* = 195)	Unique profiles (*n* = 152)	Univariate logistic regression	
	*N* (%)	*N* (%)	Crude OR (95% CI)	*p* value
Mean Age in years (SD)	35.3 (13.5)	35.6 (13.5)	1.00 (0.98–1.02)	0.868
Sex				
Male	140 (71.8)	110 (72.4)	1.00	
Female	55 (28.2)	42 (27.6)	1.03 (0.64–1.65)	0.906
Nationality				
Brazilian	160 (82.1)	106 (69.7)	1.00	
South American migrant	35 (17.9)	46 (30.3)	0.50 (0.30–0.83)	0.008
Education in years				
0–3	8 (4.9)	12 (10.0)	1.00	
4–7	52 (31.7)	36 (30.0)	2.17 (0.80–5.83)	0.126
8–11	81 (49.4)	50 (41.7)	2.43 (0.93–6.36)	0.070
12+	23 (14.0)	22 (18.3)	1.57 (0.54–4.56)	0.409
Worker				
Yes	140 (77.3)	109 (77.3)	1.00	
Retired/housewife	12 (6.6)	11 (7.8)	0.85 (0.36–2.00)	0.708
Unemployed	24 (13.3)	19 (13.5)	0.98 (0.51–1.89)	0.960
Prisoners	5 (2.8)	2 (1.4)	1.95 (0.37–10.22)	0.431
PTB incidence^a^ in district of residence				
< 40	64 (32.8)	47 (30.9)	1.00	
40–80	74 (38.0)	55 (36.2)	0.99 (0.59–1.65)	0.963
> 80	57 (29.2)	50 (32)	0.84 (0.49–1.43)	0.515
Case type				
New	145 (75.1)	117 (80.1)	1.00	
Retreatment/relapse	48 (24.9)	29 (19.9)	1.33 (0.73–2.25)	0.277
Sputum smear				
Negative	46 (24.3)	25 (16.7)	1.00	
Positive	143 (75.7)	125 (82.3)	0.62 (0.36–1.07)	0.086
Diagnosis	184 (94.4)	140 (92.7)	1.00	
Passive	11 (5.6)	11 (7.3)	0.76 (0.32–1.81)	0.535
Active case finding				
Drug resistance				
No	158 (89.8)	122 (85.9)	1.00	
Yes	18 (10.2)	20 (14.1)	0.69 (0.35–1.37)	0.294
Treatment outcome				
Cure	128 (70.7)	106 (73.1)	1.00	
Loss of follow-up	41 (22.7)	30 (20.7)	1.13 (0.66–1.94)	0.651
Death/failure	12 (6.6)	9 (6.2)	1.10 (0.45–2.72)	0.829
Household contacts				
0	16 (14.7)	11 (11.5)	1.00	
1–2	41 (37.6)	33 (34.4)	0.85 (0.35–2.09)	0.730
3–4	19 (17.4)	24 (25.0)	0.54 (0.20–1.44)	0.222
5+	33 (30.3)	28 (29.2)	0.81 (0.32–2.03)	0.653
Treatment delay in weeks				
0–2	12 (16.0)	14 (23.7)	1.00	
3–4	24 (32.0)	11 (18.6)	2.55 (0.89–7.27)	0.081
5–11	15 (20.0)	17 (28.8)	1.03 (0.36–2.91)	0.956
12 or more	24 (32.0)	17 (28.8)	1.65 (0.62–4.43)	0.323
Alcohol abuse				
No	166 (85.1)	131 (86.2)	1.00	
Yes	30 (14.9)	21 (13.8)	1.09 (0.59–2.00)	0.781
Drug use				
No	154 (79.0)	135 (88.8)	1.00	
Yes	41 (21.0)	17 (11.2)	2.11 (1.15–3.89)	0.016
TB/HIV co-infection				
No	126 (70.8)	106 (79.1)	1.00	
Yes	52 (29.2)	28 (20.9)	1.56 (0.92–2.65)	0.097
Diabetes				
No	189 (96.9)	143 (94,1)	1.00	
Yes	6 (3.1)	9 (5.9)	0.50 (0.18–1.45)	0.204

The percentage in brackets is calculated based on non-missing data. The difference between the total number of Brazilians, South American migrants or Total and each variable category corresponds to missing data

^a^Incidence per 100,000 person years

### Sensitivity analysis

When we considered only identical patterns as clustered, we found a lower proportion clustered in both populations, but the OR for belonging to a cluster in South American migrants vs Brazilians remained consistent with the main analysis (OR = 0.56, 95% CI 0.33–0.94; Table 3, rows for Clusters). The proportion of mixed clusters decreased to 12.3% of all recent transmitted cases and to 6.3% of all the cases sampled.

**Table 3:** Distribution of individuals by origin in clusters: sensitivity analysis with clusters restricted to those with identical patterns and excluding TB high-risk groups

Cluster proportion		South American migrants	Brazilians	Overall		
		*N* (%)	*N* (%)	*N* (%)	OR (95% CI)	*p* value
Identical patterns	Clusters	26 (32.1)	122 (45.9)	148 (42.7)	0.56 (0.33–0.94)	0.029
	Unique profiles	55 (67.9)	144 (54.1)	199 (57.3)		
Similar patterns, excluding HIV, drug users and alcohol abusers	Clusters	23 (33.18)	51 (42.9)	74 (39.6)	0.68 (0.37–1.27)	0.225
	Unique profiles	45 (66.18)	68 (57.1)	113 (60.4)		

After removing TB cases from high-risk groups for TB (PLHIV, drug users and alcohol abusers), 119 Brazilians and 68 South American migrants remained in our sample. The proportion of clusters decreased from 60.2% to 42.9% among Brazilians and from 43.2% to 33.8% among South American migrants (OR = 0.68, 95% CI 0.37–1.27) (see Table 3, rows for Clusters). Six mixed clusters remained. The proportion of recent transmission involved in mixed clusters increased from 24.6% to 25.7% (19/74), which corresponded to 10.2% of overall cases in our sample.

## Discussion

Our results suggest that TB disease following recent transmission in central areas from São Paulo is more common among Brazilians. Also, we suggest that cross-transmission between migrants and Brazilians is present; however, it is limited in both directions, i.e. from migrants to Brazilians and vice versa. These areas of São Paulo concentrate vulnerable populations for TB infection including a significant number of recently arrived migrants [21, 25].

In our study, 56.2% of cases of people with TB were possibly involved in clusters suggesting recent transmission. The proportion of clusters was smaller among South American migrants compared to Brazilians and higher among drug users. One out of four cases involved in recent transmission contained both Brazilians and South American migrants (mixed clusters). In most mixed clusters there was a predominance of Brazilians, with only one cluster with more South American migrants than nationals. In both our sample and in the study area, Brazilians and South American migrants differ in sociological, demographic and clinical characteristics: South American migrants tend to be younger, have higher levels of education, tend to be female and do not use drugs or carry HIV, reflecting the characteristics of healthy labour migration in the context of South America [13, 21].

The overall clustering proportion found in our study was similar to that found in high-income countries [4, 28, 29], but lower than in middle- and low-income countries with a high TB burden [30, 31]. Other studies carried out in Brazil found less than 34% of cases due to recent transmission [32]. In our sample, South American migrants were less likely to belong to a cluster than Brazilians. These results differ from those in Iran with relapse cases [11] and agree with studies conducted in high-income settings, where in general there are higher proportions of clustering among the local-born population [4]. Nevertheless, being Brazilian or being a drug user was not independently associated with clustering. This is a likely reflection of the differences between South American migrants and Brazilians regarding social and demographic characteristics and the lower proportion of comorbidities among migrants.

The incidence ratio between migrants and the local born population are generally higher in South-North migration than in South-South. Studies in high- and middle-income countries have found similar proportions of cross-transmission to those in our study, around 30–40% [4, 11], suggesting a limited impact of cross-transmission on TB burden. When we removed all individuals belonging to high-risk groups for TB resistance (drug users, PLHIV and alcohol abusers), which were predominantly Brazilians, the proportion of recently transmitted cases among South American migrants and Brazilians became similar. On the other hand, the mixed clustering proportion was unchanged. Our results add to growing evidence from low- and middle-income countries (LMICs) that belonging to these high-risk groups might still be an important factor for recent TB transmission among Brazilians [30, 33, 34].

The key limitations of our study are the short study duration and limited sampling frame. Combined with insufficient epidemiological information on contact tracing, this prevents us from assigning a likely source case. However, in larger clusters, for example where only one out of nine or more cases is from a different origin group, transmission was most likely to this individual, rather than in the opposite direction. This could suggest that cross-transmission from Brazilians to migrants is more likely than that from migrants to Brazilians, which would be in line with other studies where a majority rule is used to designate the origin of the ’primary’ case in that cluster [7, 12]. Furthermore, variables such as country of origin and time since arrival in Brazil (for migrants), which could add more information to TB incidence in the country of origin and to the risk of clustering, were not available in the Brazilian notification system when this study was conducted. More studies are needed to estimate the most likely direction of TB transmission, in order to study the characteristics and measure the impact of migration in our setting.

The short duration and low sampling proportion are also likely to lead to an underestimation of the clustering in both populations [6, 27] in potentially equal measure. However, due to the oversampling of Brazilian drug users and PLHIV, who are more likely to be part of a cluster, clustering is probably overestimated in Brazilians. As migrants are considered a priority group for sputum culture in the study area, our sample provided a more accurate estimate of transmission in migrants. We therefore suspect that underestimation of clustering among South American migrants is more likely than among Brazilians, which could mean that the contribution of mixed clusters to ongoing transmission in the study area is higher than found here [6]. Cross-contamination must always be considered in a unique cluster of migrants with only one Brazilian, although we observed strict protocols during the collection of molecular characterization data.

Because of these sampling biases, we should consider this study exploratory, and it reflects the challenges of conducting molecular epidemiological studies in low- and middle-income settings. Another possible limitation of the study is the use of RFLP instead of whole genome sequencing (WGS). This could have provided lower estimates of recent transmission, especially if strain variability in countries of origin were low [35], although it is unlikely that RFLP overestimated cross-transmission. Also WGS would have provided more information on the most likely direction of transmission. Nevertheless, the existence of mixed clusters provides strong evidence for the existence of cross-transmission and the need to explore the direction and more precise estimates of the contribution of migration to the transmission of TB in LMICs.

## Conclusions

This study contributes to our understanding of the influence of South-South migration on TB recent transmission and cross-transmission in the central area of São Paulo. Marked social inequalities in middle-income countries and in the context of regional migration must be considered to reach the End TB Strategy targets. TB care and prevention policies should contemplate the characteristics of migration and the living conditions in host countries, as has been done in high-income countries [36], and target those groups, both migrants and local-born populations, in which recent transmission is more evident.

## Additional file

Additional file 1: Supplement **Table S1** describe the cases of TB among Brazilians and South American migrants in the study area. Some differences regarding demographic characteristics and TB risk factors for TB transmission were less pronounced in the study area compared to the study group, suggesting oversampling of retreatments, PLHIV and drug users among Brazilians. (DOCX 17 kb)

## Abbreviations

IAL-SP: Instituto Adolfo Lutz-São Paulo
*Mtbc*: *Mycobacterium tuberculosis* complex
PLHIV: People living with HIV
PTB: Pulmonary tuberculosis
RFLP: Restriction fragment length polymorphism
SINAN-TBWEB: São Paulo Tuberculosis Control Program database
TB: Tuberculosis
WHO: World Health Organization

## Footnotes

1 The population considered near poor in a monetary sense has an equivalent income of more than $1.25 a day but less than $2.50 a day [23].
