# Supplementary material for: Migration and tuberculosis transmission in a middle-income country: a cross-sectional study in a central area of São Paulo, Brazil
Source: BMC Med. 2018 Apr 30;16:62. doi: 10.1186/s12916-018-1055-1 (PMC5925834; doi:10.1186/s12916-018-1055-1)
Supplement: Supplementary file 1 — Supplement Table S1 describe the cases of TB among Brazilians and South American migrants in the study area. Some differences regarding demographic characteristics and TB risk factors for TB transmission were less pronounced in the study area compared to the study group, suggesting oversampling of retreatments, PLHIV and drug users among Brazilians. (DOCX 17 kb) [file 12916_2018_1055_MOESM1_ESM.docx]

**Additional file 1.**

Supplement Table 1 describe the cases of TB among Brazilians and South American migrants in the study area. Some differences regarding demographic characteristics and TB risk factors for TB transmission were less pronounced in the study area compared to the study group, suggesting oversampling of retreatments, PLHIV and drug users among Brazilians.

Table S1 Description of cases of TB among Brazilians and South American migrants in the study area - Potential bias regarding to sample selection.

|  | **Brazilians**  **(n=1393)** | **South-American migrants (n=334)** | **Total**  **(n=1764)** |  |
| --- | --- | --- | --- | --- |
|  | **N (%)** | **N (%)** | **N (%)** | ***p-value**** |
| **Mean age (sd)** | 39.6 (16.1) | 26.5 (11.3) | 37.2 (16.3) | <0.001 |
| **Sex** |  |  |  | 0.111 |
| Male | 945 (67.8) | 212 (63.3) | 1181 (66.9) |  |
| Female | 448 (32.2) | 123 (36.7) | 583 (33.1) |  |
| **School attendance** |  |  |  | 0.790 |
| 0-3 | 92 (8.9) | 22 (8.0) | 116 (8.7) |  |
| 4-7 | 326 (31.4) | 80 (29.7) | 411 (30.9) |  |
| 8-11 | 435 (41.9) | 122 (45.4) | 566 (42.5) |  |
| 12+ | 185 (17.8) | 45 (16.7) | 239 (17.9) |  |
| **Case** |  |  |  | <0.001 |
| New | 1122 (81.3) | 301 (91.2) | 1454 (83.3) |  |
| Retreatment/relapse | 258 (18.7) | 29 (8.8) | 292 (16.7) |  |
| **Treatment outcome** |  |  |  | <0.001 |
| Cure | 893 (68.7) | 249 (81.1) | 1159 (70.8) |  |
| Loss of follow up | 285 (21.9) | 44 (14.3) | 335 (20.5) |  |
| Others | 122 (9.4) | 14 (4.6) | 143 (8.7) |  |
| **Sputum smear** |  |  |  | 0.150 |
| Neg | 330 (26.0) | 93 (30.0) | 437 (27.1) |  |
| Pos | 941 (74.0) | 217 (70.0) | 1173 (72.9) |  |
| **Drug resistance**** |  |  |  | 0.576 |
| No | 336 (88.2) | 87 (86.1) | 429 (87.9) |  |
| Yes | 45 (11.8) | 14 (13.9) | 59 (12.1) |  |
| **HIV test** |  |  |  | <0.001 |
| Neg | 917 (77.8) | 289 (97.0) | 1225 (81. 6) |  |
| Pos | 261 (22.2) | 9 (3.0) | 277 (18.4) |  |
| **Diabetes** |  |  |  | 0.026 |
| No | 1314 (94.3) | 326 (97.3) | 1671 (94.7) |  |
| Yes | 79 (5.7) | 9 (2.7) | 93 (5.3) |  |
| **Alcohol abuse** |  |  |  | <0.001 |
| No | 1174 (84.3) | 324 (96.7) | 1531 (86.8) |  |
| Yes | 219 (15.7) | 11 (3.3) | 233 (13.2) |  |
| **Drug user** |  |  |  | <0.001 |
| No | 1198 (86.0) | 327 (97.6) | 1561 (88.5) |  |
| Yes | 195 (14.0) | 8 (2.4) | 203 (11.5) |  |

The percentage in the brackets is calculated based on non-missing data. The difference between the total number of Brazilians, South American migrants or Total and each variable category correspond to missing data.

* Two tailed T-test for mean age comparison and Pearson Chi-square for categorical variables.

** Resistant to at least one drug
